# Machinability and Surface Properties of Cryogenic Poly(methyl methacrylate) Machined via Single-Point Diamond Turning

**DOI:** 10.3390/ma17040866

**Published:** 2024-02-13

**Authors:** Xiaoyu Wu, Qiang Kang, Xiaoxing Jiang, Xudong Fang

**Affiliations:** 1 School of Mechanical Engineering, Xi’an Jiaotong University, Xi’an 710049, China; xymems@stu.xjtu.edu.cn (X.W.); xxjiang_2022@stu.xjtu.edu.cn (X.J.); 2 State Key Laboratory for Manufacturing Systems Engineering, International Joint Laboratory for Micro/Nano Manufacturing and Measurement Technology, Xi’an Jiaotong University, Xi’an 710056, China; 3 School of Electrical Engineering, Xi’an Jiaotong University, Xi’an 710049, China; 4 School of Mechano-Electronic Engineering, Xidian University, Xi’an 710071, China; kangqiang@xidian.edu.cn

**Keywords:** poly(methyl methacrylate) (PMMA), mechanical property, cryogenic, single point diamond of turning (SPDT), nanoindentation, surface roughness

## Abstract

Poly(methyl methacrylate) (PMMA), with a glass transition temperature (Tg) over 100 °C, shows good mechanical and optical properties and has broad applications after being machined with single-point diamond turning (SPDT) at room temperature. Because of the high Tg, current efforts mostly focus on optimizing machining parameters to improve workpiece precision without considering the modification of material properties. Cryogenic cooling has been proven to be an effective method in assisting ultra-precision machining for certain types of metals, alloys, and polymers, but has never been used for PMMA before. In this work, cryogenic cooling was attempted during the SPDT of PMMA workpieces to improve surface quality. The machinability and surface properties of cryogenically cooled PMMA were investigated based on the mechanical properties at corresponding temperatures. Nanoindentation tests show that, when temperature is changed from 25 °C to 0 °C, the hardness and Young’s modulus are increased by 37% and 22%, respectively. At these two temperature points, optimal parameters including spindle speed, feed rate and cut depth were obtained using Taguchi methods to obtain workpieces with high surface quality. The surface quality was evaluated based on the total height of the profile (Pt) and the arithmetic mean deviation (Ra). The measurement results show that the values of Pt and Ra of the workpiece machined at 0 °C are 124 nm and 6 nm, respectively, while the corresponding values of that machined at 25 °C are 291 nm and 11 nm. The test data show that cryogenic machining is useful for improving the form accuracy and reducing the surface roughness of PMMA. Moreover, the relationship between temperature, material properties and machinability weas established with dynamic mechanical analysis (DMA) data and a theoretical model. This can explain the origin of the better surface quality of the cryogenic material. The basis of this is that temperature affects the viscoelasticity of the polymer and the corresponding mechanical properties due to relaxation. Then, the material property changes will affect surface profile formation during machining. The experimental results and theoretical analysis show that cryogenically cooled PMMA has good machinability and improved surface quality when using SPDT compared to that at ambient temperature.

## 1. Introduction

Single point diamond turning (SPDT) is one of the dominant processes used when producing ultra-precision parts. It can be used for various materials, including metals, ceramics, and polymers. Using this process, workpieces with a designed geometry and specific properties can be obtained for multiple applications like the aerospace industry, the automobile industry, etc. In recent decades, researchers have exerted great efforts in studying the effect of machining parameters, including the feed rate, spindle speed and cut depth, on component surface quality. Plenty of work has been completed on metals. For example, stainless steel, aluminum, and copper are processed using SPDT to reach micro-/nano-level accuracy with optimized machining conditions [1,2]. Nevertheless, investigations on the SPDT of polymers are quite limited. Only a few types of polymers can be machined using this process [3,4]. The hardness and Young’s modulus of the polymers must reach a certain value to use SPDT; otherwise, the accuracy of the final part cannot be reached because of deformation induced by softness. A representative of the polymers which cannot be directly machined using SPDT is PDMS, which is too soft to maintain dimensional precision. Accordingly, the ultra-precision machining of polymers via SPDT is still a hot topic requiring further research.

PMMA, as a typical example of a polymer with excellent optical properties, has been used in many applications after being machined using SPDT. Its applications include biomedical instruments, consumer products, communication systems, and security instrumentation, depending on the material properties of PMMA, including its rigidity, toughness, light transmission, dimensional stability, and surface quality. Among the aforementioned properties, the surface quality of PMMA is a major challenge to address to extend its use to broader application fields. Researchers have attempted to use Taguchi orthogonal methods to optimize the machining parameters such as the depth of the cut, the spindle speed, the tool feed rate, the tool nose radius, and the coolant in order to obtain a high surface quality [5,6,7,8]. Bhaskar Goel et al. reported that the prominent factors affecting surface roughness were spindle speed, tool overhang and the feed rate of cutting, while the key factors affecting profile and waviness errors were spindle speed, cut depth and tool nose radius [9]. K. Jagtap et al. studied the influence of cutting parameters on the surface quality of PMMA obtained via SPDT [4]. They found that spindle speed is a major factor affecting surface flatness in the SPDT of PMMA. However, in the abovementioned published literature studying the effects of machining parameters, all the experiments were executed at room temperature, and the achievable minimum PV value was only about 0.83 µm, which needs to be lower in order to meet application requirements of PMMA optical components. 

To further improve the surface accuracy of PMMA parts produced using SPDT, in addition to the machining parameters, the material properties need to be adjusted. The crystalline structure, glass transition temperature and viscoelasticity can cause significant form errors in the machined parts [10]. Yasuhiro Kakinuma and collaborators applied cryogenic cooling to soft PDMS in the cutting process to machine microgrooves [11]. They successfully changed the material from a rubbery state to a glassy state below the glass transition temperature in order to obtain a remarkable change in elasticity. With this material property change, an accurate shape can be obtained easily. Similar methods have been used for other materials to keep the temperature below their glass transition temperature in order to increase their hardness for machining [12,13,14]. The cryogenic temperature ensures that the polymeric material is ductile and elongational and enables it to withstand cutting forces without deformation. 

Cryogenic cooling has been proven to be one effective way to change the material properties of polymers to make them suitable for ultra-precision machining. By quenching them below the glass transition temperature, their hardness can be considerably improved to avoid flexible deformation during machining. This method has been demonstrated by many researchers for soft polymers like PDMS and EVA. However, it has not been used for the SPDT of PMMA. One possible reason for this is that the glass transition temperature of this polymer is over 100 °C, far beyond ambient temperature. The material is in a glassy state when machining, and thus does not need further cooling. So far, no reports are available about the material property changes of PMMA for SPDT and the corresponding effects on the surface quality of the final components. 

To obtain high-precision PMMA optical components with a superior surface quality, this work aims at changing the material properties of PMMA via SPDT with cryogenic cooling and investigating the effects on the machinability and surface quality of the final part. If it succeeds, many high-precision, lightweight parts with a smooth surface can be manufactured using PMMA, which can be widely used in aerospace, aviation, etc. PMMA was maintained at a temperature 100 °C lower than the Tg during machining. The Taguchi method was used to change the spindle speed, feed rate and depth of the cut to search for the optimal conditions for machining a flat surface. In addition, nanoindentation tests were performed to check the hardness and Young’s modulus of the PMMA at different temperatures. The purpose was to obtain the relationship between the temperature, material properties, processing conditions and surface quality in order to fabricate PMMA components with high precision. 

## 2. Experimental

### 2.1. Materials

PMMA materials were supplied by Mitsubishi Chemical Holdings (Beijing, China) Co., Ltd. Workpieces were obtained by means of turning using a lathe to a size of Ø10 mm × 10 mm. Cryogenic samples for nanoindentation tests were obtained by soaking the workpieces in liquid nitrogen for 5, 10, 15 or 60 min, while the workpieces for SPDT were cooled by flushing liquid nitrogen from a nozzle during machining.

### 2.2. Temperature Variation Test

In the tests regarding the hardness and Young’s modulus of the materials, as the PMMA samples could not be maintained at a very low temperature during the tests due to the restrictions of the instrument, it was necessary to monitor the temperature on a flat surface by checking the time. The temperature as a function of time was obtained using the following procedure. The samples were soaked in liquid nitrogen for a certain time, then taken out and exposed to the air. The temperature shift with time on the flat surface was measured using an AT4516 multichannel temperature meter (Applent Instrument, Edison, NJ, USA). 

### 2.3. Nanoindentation Test

First, the workpieces were polished using Mecatech 234 (PRESI, Paris, France) to obtain a relatively flat and smooth surface for test accuracy. Then, they were soaked in liquid nitrogen for 5, 10, 15 and 60 min before being taken out for nanoindentation. Hysitron TI950 (Bruker, Billerica, MA, USA) was used to test the mechanical properties of PMMA in a micro view for the hardness and Young’s modulus. The indenter had a Berkovich tip with 142.3° incl. angle and 100 nm R with a standard holder. To save time and reduce test cost, an indentation depth of 1µm was selected to ensure elastic deformation by referring to the literature [15]. As the hardness and Young’s modulus of PMMA are sensitive to strain rates, following one recent published paper, a strain rate of 0.2/s was used to obtain a stable value of the Young’s modulus [16]. If the rate was lower than this value, the Young’s modulus among the tests would have a large variation. Tests were completed for the samples with a surface temperature of 0, 5, 10, 15, 20 and 25 °C. At each temperature, 5 points at different locations on each sample were tested to obtain the average value. With this method, the mechanical properties of PMMA as a function of temperature could be obtained. 

### 2.4. SPDT of Cryogenically Cooled PMMA

**Figure 1:**
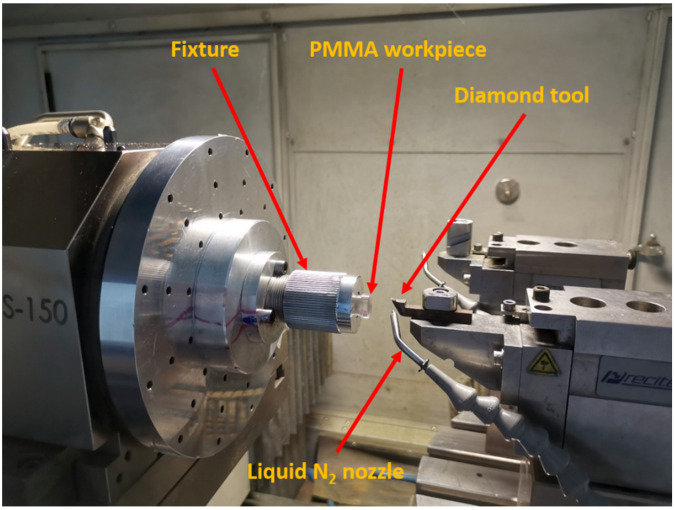
Setup for the SPDT of PMMA with cryogenic cooling.

Nanoform 700 ultra (Precitech, Keene, NH, USA) was used to complete SPDT of the workpieces with dimensions of Ø10 mm × 10 mm on the flat surface. They were divided into two groups: one group machined at room temperature as the control samples, and the other group machined at a low temperature by cooling with liquid nitrogen. The workpieces were fixed on the spindle using an air chuck and were rotated at the speed of the spindle. Machining parameters including the spindle speed, depth of the cut and the feed rate could be set with the movement of the *x*-axis and *z*-axis of the machine. The diamond tool used had a 0.491 mm radius, a 0° rake angle, and a 10° clearance angle. The setup for the SPDT of PMMA is shown in Figure 1. 

### 2.5. Measurement and Characterization Tools

**Figure 2:**
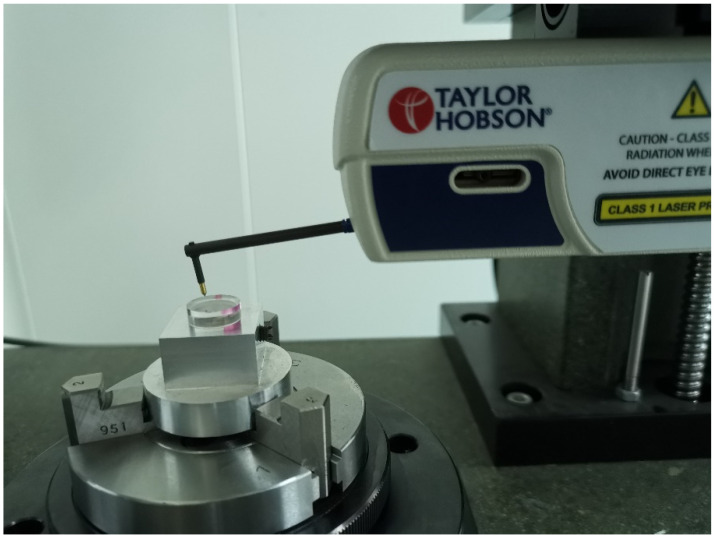
Setup of PMMA workpiece measurement with a Taylor Hobson device.

A Taylor Hobson device (Ametak, Leicester, UK) was utilized to measure the form profile and surface roughness of the machined flat surface with the contacting method. A specific holder was designed to fix the PMMA workpiece during the test. The measurement setup is shown in Figure 2. 

The viscoelasticity of PMMA was tested using DMA. A standard bar of PMMA with dimensions of 7.3 × 7.4 × 2.0 mm was tested following the frequency sweep and temperature sweep procedures using DMA 8000 (PerkinElmer, Waltham, MA, USA). With the frequency sweep as the first step, the frequency range for the linear viscoelastic region can be obtained. Then, at a fixed frequency of 1 Hz, a temperature sweep from 25 °C to −70 °C was completed. An oscillating force was applied and the resulting Young’s modulus and tanδ could be calculated.

## 3. Results and Discussion

### 3.1. Temperature Change of Cryogenically Cooled PMMA in Air

**Figure 3:**
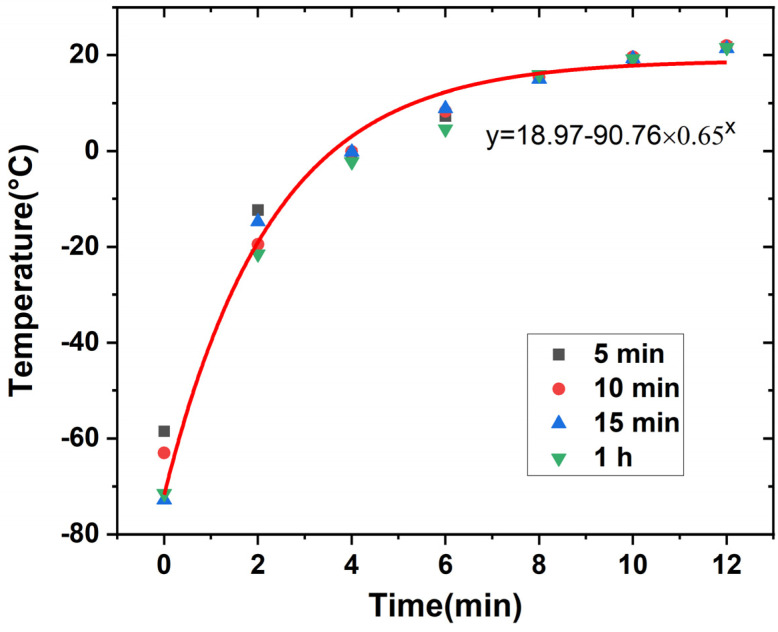
Temperature change of cryogenically cooled PMMA with time when exposed to air.

No tool or setup is available to maintain the materials at a low temperature during the nanoindentation tests. Thus, the tests were generally completed at room temperature. The purpose of this experiment was to check the temperature shift of the material in air to determine the surface temperature at a specific time. Then, the material properties including the hardness and Young’s modulus could be related to surface temperature by monitoring the time when the test was executed. Accordingly, the effect of material property change on the machinability of PMMA using SPDT could be investigated. This temperature shift curve is the basis for further studies on cryogenically cooled PMMA. The workpieces were soaked in liquid nitrogen for 5, 10, 15 or 60 min to test the temperature ramp rate in air. As observed in Figure 3, the workpieces could be cooled to −60 °C or even −70 °C. After they were exposed in air, the temperature on the surface increased to room temperature within around 12 min. Analysis of the data proves that the soaking time has a small effect on the temperature ramp rate. The samples with a soaking time of over 5 min show quite similar temperate shift curves, which can be expressed with the following fitted equation.

*y* = a − bc*^x^* (1)

In Equation (1), *y* is the temperature on the workpiece surface and *x* is the time of exposure to air. a, b, and c are constants that are obtained by means of nonlinear fitting. In Figure 3, a fitting curve for 15 min is provided as an example. With this plot, the temperature in nanoindentation tests and other types of hardness tests for PMMA workpieces can be calculated by monitoring the time elapsed since leaving the liquid nitrogen bath. 

### 3.2. Mechanical Property of Cryogenically Cooled PMMA

Examples of using the nanoindentation test to check the hardness and elastic modulus of PMMA and study the effects of loading rate, holding time, etc., can be found in the literature. However, all of the published experimental results were obtained at an ambient temperature. One possible reason for this phenomenon is that major challenges exist regarding testing at very low temperatures, including possible damage to the indentation tip and the whole instrument. Nevertheless, the mechanical properties at temperatures far below the Tg are very important for studying the structure, performance and processing of PMMA. Hence, efforts need to be made to investigate the hardness and elastic modulus of cryogenically cooled PMMA. The samples were soaked in liquid nitrogen for 15 min and then removed for testing. The tips were compressed into samples when the surface temperature was 0, 5, 10, 15, 20 and 25 °C by monitoring the time in the air. As the total process, including uploading, holding and unloading, was completed in about 15 s, the temperature was assumed to be constant during the process. 

**Figure 4:**
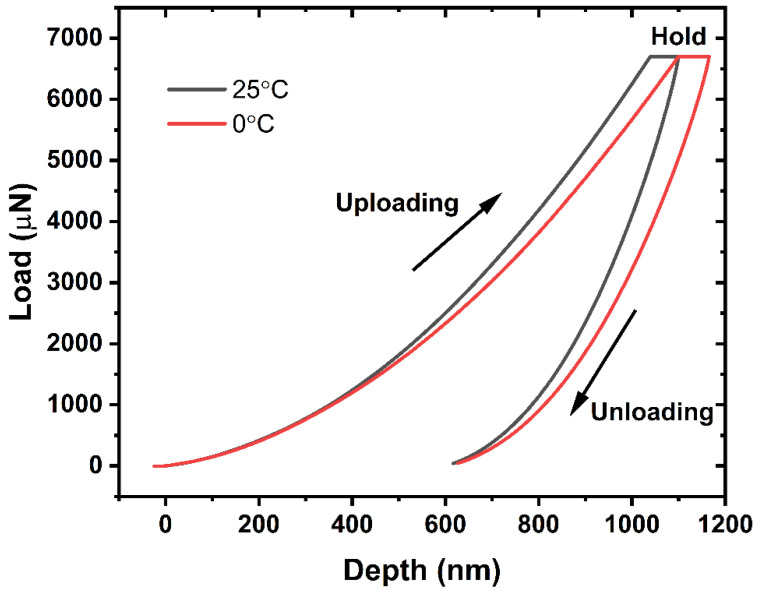
Representative load–depth curves of PMMA at 25 °C and 0 °C.

Typical load–depth curves of PMMA at 25 °C and 0 °C are shown in Figure 4. A maximum load of 6500 µN with a load rate of 1300 µN/s was applied to reach a depth of 1 µm to calculate the hardness and elastic modulus. At each temperature, indentation tests were repeated five times to obtain the average value of the target parameters. The indentation tests were performed five times on the exact same sample, including soaking in liquid nitrogen and measurement at a definite point. The soaking time and corresponding temperature were calculated based on the relationship obtained in Figure 3. 

**Figure 5:**
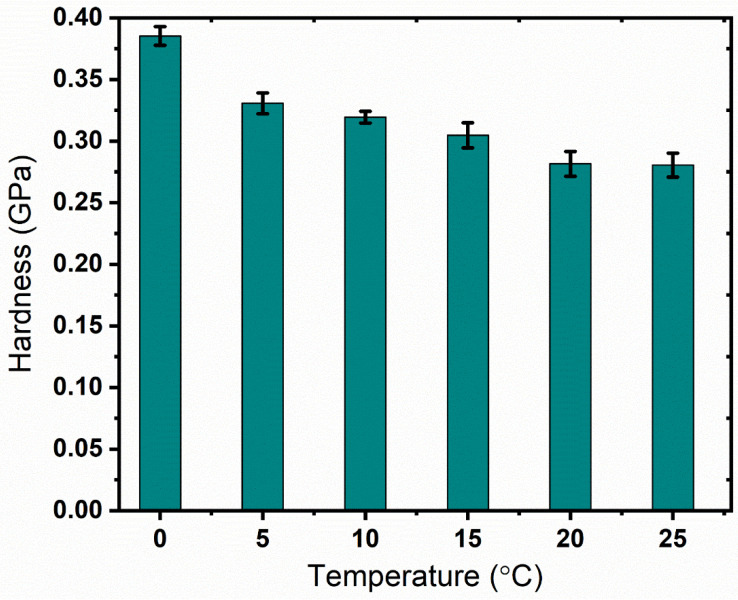
Nanoindentation hardness value of PMMA at different temperatures.

**Figure 6:**
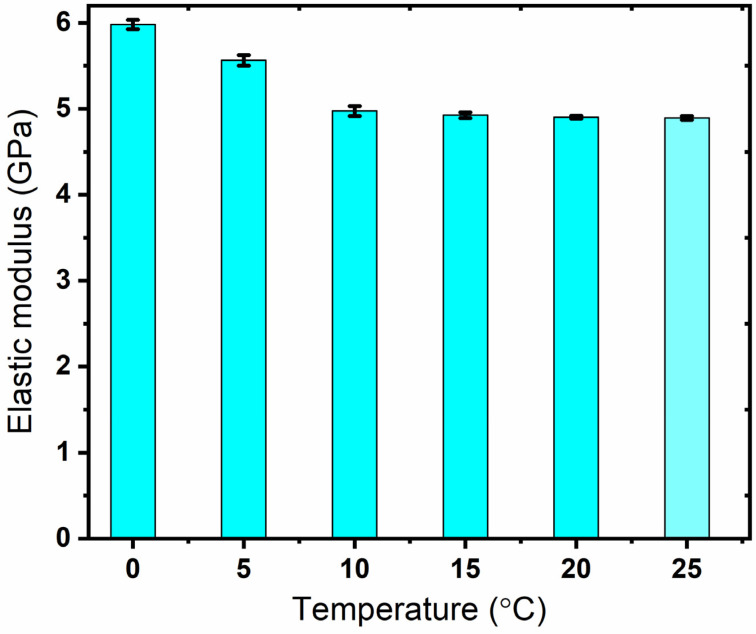
Nanoindentation elastic modulus value of PMMA at different temperatures.

The average hardness and elastic modulus at the above temperatures are shown in Figure 5 and Figure 6, respectively. As observed from the figures, the test data show excellent repeatability with small standard deviations. This is attributed to the homogenous microstructure and the maintenance of a stable state at temperatures far below the Tg. The nanoindentation hardness and elastic modulus of PMMA continuously increase when lowering the sample temperature by controlling the initial time of indentation. In particular, the hardness is significantly affected by temperature. It increases by 37% when the surface temperature is reduced from 25 °C to 0 °C. Meanwhile, the elastic modulus is increased by 22% when the temperature shifts from 25 °C to 0 °C.

**Figure 7:**
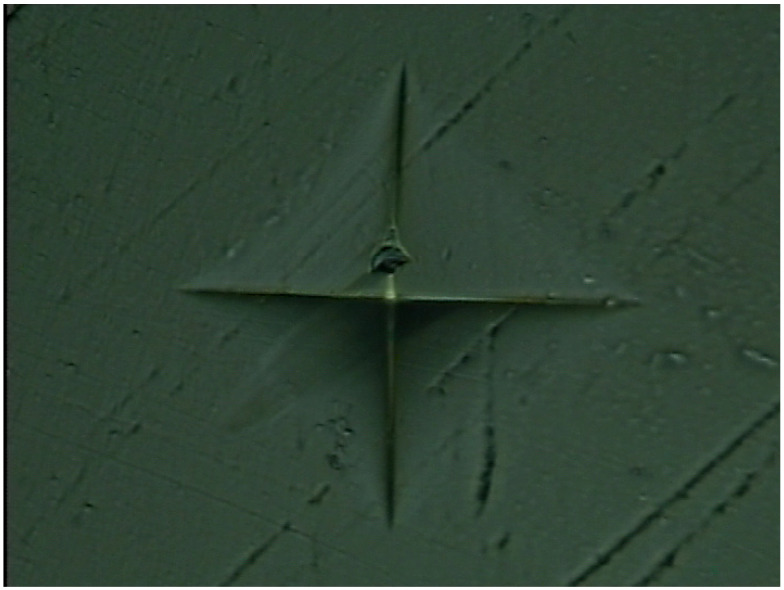
One typical indentation point of cryogenically cooled PMMA under a microscope.

As no tests for mechanical properties of cryogenically cooled PMMA are available in the literature, the hardness of the samples was retested with a micro hardness tester for authenticity. A micro hardness tester from Shanghai Shuming Optical Instrument (HXD-1000 TMC/LCD) (Shanghai, China) was used to check the HV values of the cryogenic samples. The HV values at the same temperature as tested in the nanoindentation tests are listed in Table 1. As obtained from Table 1, the HV value decreases with an increase in temperature. This is attributed to the material properties of PMMA, which is an amorphous polymer with viscoelasticity, as described in Section 3.4. Meanwhile, the hardness is positively related to the storage modulus. Accordingly, this is consistent with the result in Figure 12, showing that the ratio of the loss modulus to the storage modulus increases with temperature from 0 to 25 °C, which means that the hardness will show a decreasing trend following an increase in temperature. As observed in Figure 5, the unit of hardness obtained in the nanoindentation tests is GPa, while that obtained with the micro hardness tester is kgf/mm^2^. The hardness decreases with increase in temperature, displaying the same trend as in the nanoindentation tests. In addition, the data show good repeatability with small errors, indicating uniform surface properties. After being converted to the same units, the values at the same temperature obtained with the two methods are different. One interesting phenomenon is that the difference is almost constant for the values at the five temperature points. The difference between the hardness value obtained with the micro hardness tester and that obtained from the nanoindentation tests was calculated with the same unit (GPa). The difference between the values at the five temperature points obtained with the two test methods was also calculated. For example, at 0 °C, the hardness obtained with the micro hardness tester was HV30.2 (0.29596 GPa), while that obtained with the nanoindentation tests is 0.38527 GPa, with the difference being 0.08931 GPa. The difference at the five temperature points was calculated and the average difference was 0.087965 GPa with a standard deviation error of 0.01030645 GPa. One possible reason for this is that a system error remains in the two test methods during measurement unit conversion. A representative indentation point of the PMMA produced by the micro hardness tester under a microscope is shown in Figure 7. The indented profile as observed in the figure is clear and large, which indicates that the hardness is low. We observed the trend that the harder the material is, the smaller the indented area. If the material is super rigid, the indented area will only cover one small area, like a point. The hardness value (HV) is also calculated based on the force applied and the cross-sectional area of the indented region. Hence, as observed from Figure 7, the PMMA material has a relatively low hardness. Comparing the data obtained using the two test methods, it can be confirmed that mechanical properties of PMMA are enhanced by lowering the temperature. Both the hardness and the elastic modulus experience a noticeable increase when the temperature is changed from 25 to 0 °C.

### 3.3. Machinability and Surface Property of Cryogenically Cooled PMMA

PMMA is widely used in optical components in various fields requiring a high level of precision, and is commonly processed using SPDT. Thus, to meet the increasing demand for components with higher accuracy, it is important to study the processing methods of PMMA for higher precision and efficiency. In this work, liquid nitrogen cooling was applied to further investigate the machinability of cryogenically cooled PMMA using SPDT, which causes the temperature of the workpiece’s surface to be 100 °C lower than the material’s Tg. The setup for the SPDT of PMMA is displayed in Figure 1. A simple flat surface was machined in the SPDT experiments for the comparison of accuracy and efficiency, while a group of workpieces machined using SPDT at room temperature were used as references. 

Machining parameters including the spindle speed, feed rate and depth of the cut were tested in groups following the Taguchi orthogonal method at the designed temperature to obtain a high surface quality. For the design of the experiment with the Taguchi method, according to the authors’ pilot study in SPDT and the reported parameters in the literature, three input parameters including the spindle speed, feed rate, and cut depth were identified as factors and three levels of each factor were chosen for the surface quality study. The factors with the corresponding levels are listed in Table 2. The surface roughness (Ra, nm), and profile error (Pt, nm) were selected as the principal response parameters, which were measured via a Taylor Hobson device using the setup shown in Figure 2. To save time and reduce cost, only one temperature point, 0 °C, was used as a demo of cryogenically cooled PMMA for SPDT. This temperature was controlled by the adjusting flow rate of liquid nitrogen and was measured in real time using an infrared thermometer.

**Figure 8:**
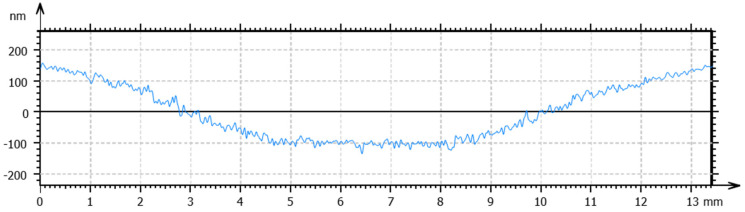
Surface profile of the PMMA workpiece machined via SPDT at room temperature under optimal processing conditions.

**Figure 9:**
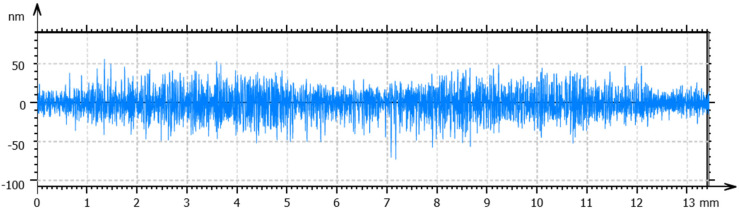
Surface roughness of the PMMA workpiece machined via SPDT at room temperature under optimal processing conditions.

**Figure 10:**
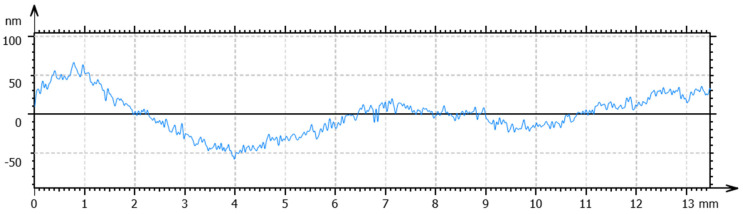
Surface profile of the PMMA workpiece machined via SPDT at 0 °C under optimal conditions.

**Figure 11:**
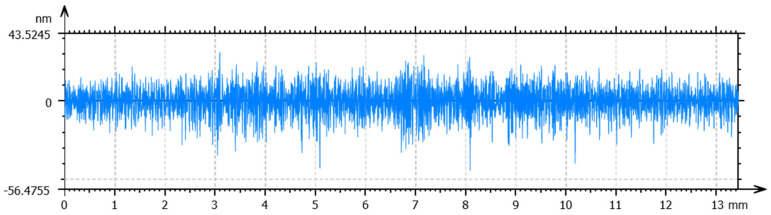
Surface roughness of the PMMA workpiece machined via SPDT at 0 °C under optimal conditions.

Optimized processing conditions, including spindle speed, feed rate and cut depth, were obtained for the specified temperature to obtain a high surface quality. The total height of the profile (Pt) and the arithmetic mean deviation (Ra) were used to evaluate form accuracy and surface roughness, respectively. For the workpieces machined at room temperature, a spindle speed of 1500 rpm, a feed rate of 5 mm/min, and a cut depth of 4 µm obtained the lowest values of Pt and Ra. The measured form accuracy and surface roughness are shown in Figure 8 and Figure 9, respectively. As observed in the figures, a Pt value of 291 nm and an Ra of 11 nm were obtained. Following the same procedure, the optimal processing conditions for the SPDT of cryogenically cooled PMMA at 0 °C are a spindle speed of 2500 rpm, a feed rate of 10 mm/min and a cut depth of 2 µm. The form accuracy and surface roughness are shown in Figure 10 and Figure 11, respectively. A Pt value of 124 nm and a surface roughness of 6 nm were obtained. Compared to the values at room temperature, Pt was decreased by 57.4%, and Ra was about 50% lower. It can be inferred that cryogenic machining is beneficial for improving the surface quality of PMMA. As the Tg of PMMA is around 105 °C, this work was completed at a temperature far below the Tg (>100 °C), which has not been achieved before. One reason for the initiation of this work was to investigate the effect of cryogenic cooling on machinability and surface quality for materials like PMMA with a high Tg. As observed from the results, the cryogenically cooled PMMA can be machined far below its Tg. With cryogenic cooling, a higher surface quality with low values of Pt and Ra can be obtained. Meanwhile, it is found that a higher spindle speed, a higher feed rate, and a smaller cut depth are needed to obtain a better surface quality at low temperatures. 

### 3.4. Relationship between Temperature, Material Property and Machinability

**Figure 12:**
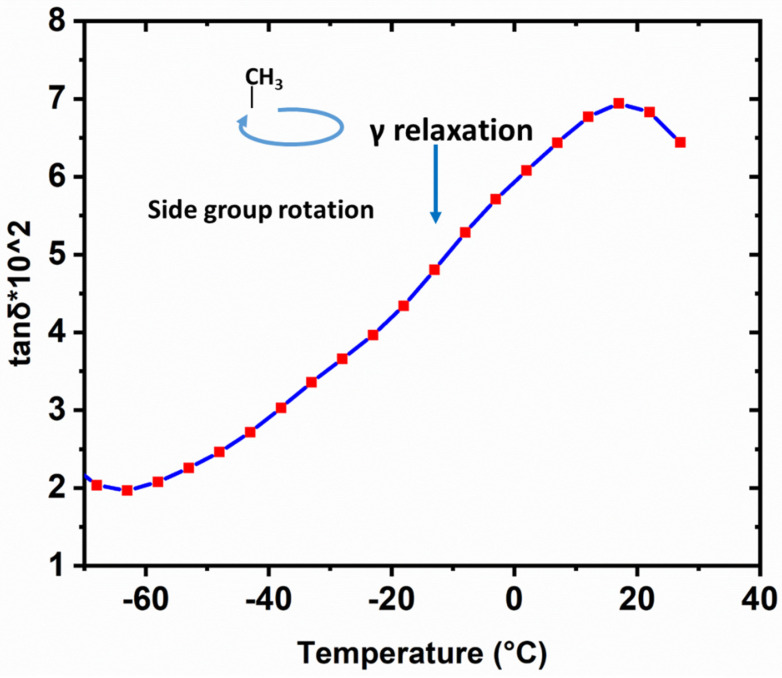
tanδ of PMMA measured at a fixed frequency of 1 Hz from 25 °C to −70 °C using DMA8000.

To understand the mechanism of the improved surface quality of cryogenically cooled PMMA using SPDT, the relationship between the material properties and the machined surface quality is investigated, with temperature as the connection. At low temperatures, the hardness and Young’s modulus are significantly increased compared to those at room temperature. Accordingly, a better surface quality with lower values of Pt and Ra can be obtained. There is an inherent connection between them. PMMA, as an amorphous polymer, has typical viscoelastic properties, considerably affecting its mechanical properties and machinability. Thus, its viscoelasticity was studied using DMA. Tanδ is the ratio of the loss modulus to the storage modulus, and its change with temperature is shown in Figure 12. One peak is available around 20 °C, which has a high possibility of representing β relaxation attributed to the side group motion of (—COO—CH_3_) [17]. With temperature decreasing from the peak point, γ relaxation mainly occurs with the rotation of the side group (—CH_3_), possibly requiring some local main chain motion [18]. These are quite different from α relaxation, which describes large-scale cooperative rearrangements of the molecules in the region of glass transition. The glassy state relaxation behavior (α, β, and γ relaxation process) is closely related to the mechanical properties of polymers [19,20]. In this work, cooling from room temperature shows a typical suppression of the sub-Tg relaxation process. With a temperature decrease, the number of segments participating in the β relaxation (i.e., the number of segments dissipating mechanical energy) reduces and the transition into γ relaxation will be reflected in macroscopic properties such as the hardness and Young’s modulus, as demonstrated in the nanoindentation tests. 

The temperature is 100 °C lower than the Tg, which is sufficient to obtain a non-equilibrium glassy state and maintain the orientation of the chain segments during machining. The α and β relaxation, which can be explained with free volume theory [21], describes the molecular change of the polymer, which can provide guidance for SPDT. At temperatures far below the Tg (Tg-100), no significant change in defect concentration occurs according to the quasi-point defect model. However, the molecular mobility of PMMA is significantly decreased, as verified by the low-temperature part of the γ relaxation. The loss of chain mobility is mainly caused by local molecular rearrangements [22]. This reduction in chain mobility increases the stiffness of the workpiece, thus avoiding detrimental deformation. 

Cryogenic cooling far below the Tg is beneficial for the improvement of mechanical properties, and its effect on the machinability of PMMA is also analyzed through surface profile modelling in SPDT. In diamond turning, the surface profile is commonly modelled per feed. As reported in previous studies, the profile is mainly affected by three components: the duplication effect of the diamond tool edge profile, material spring back and material plastic side flow [23,24]. Accordingly, the vertical distance between the highest peak and the lowest valley, i.e., F(x), can be expressed as

(2) $$F\left(x\right)=R_{tew}\left(x\right)+\frac{4(w_{r}-s_{r})}{f^{2}}(-\frac{f}{2}\leq x\leq \frac{f}{2})$$

where *Rtew*(*x*) is the active tool edge profile with consideration of tool edge waviness rate; *w_r_* is the value of material plastic side flow in the center (*x =* 0), and *s_r_* is the material spring back at the margin positions (*x* = *±f*/2) in one rate [25]. 

The surface profiles in this work can be analyzed using this empirical model. In Equation (1), *Rtew*(*x*) is determined by the tool corner nose radius and edge waviness [26]. During the SPDT of PMMA in this work, the same tool was used. Therefore, this factor can be considered the same when comparing the profiles. The material plastic flow *w_r_* is relatively complex and has insufficient calculation methods to obtain an analytical solution [25]. Nevertheless, for PMMA, it is mainly affected by the material’s viscoelasticity. There exists a positive correlation between the plastic flow and the viscous behavior. For a qualitative comparison, according to the tanδ values at 0 °C and 25 °C in Figure 12, the loss modulus plays a more dominant role when temperature is higher, which represents viscous performance. This indicates that *w_r_* will be larger at 25 °C. Meanwhile, material spring back *s_r_* can be expressed as a proportional function of the tool edge radius and the ratio of a workpiece’s material hardness to its Young’s modulus (*H/E*) [27,28]. In this work, as proven by the available test data, the value of *H/E* at 0 °C is 1.12 times greater than that at 25 °C. Thus, considering the same diamond tool and value of *H/E*, the material spring back value *s_r_* at 0 °C is larger. A comparison of the three factors at the two temperatures is summarized in Table 3. Substituting the comparison results into Equation (2), we can obtain a qualitative result indicating that the value of the surface profile at 0 °C is obviously smaller. This is consistent with the measurement results reflected by the Pt and Ra values. 

A major effect of cryogenic cooling on PMMA is the control of its viscoelasticity. Lowering the temperature can suppress the viscous behavior of the material so that the relaxation time is longer, which makes the material more rigid with a higher hardness and Young’s modulus. Meanwhile, the suppression of viscous behavior is useful for decreasing the material side plastic flow during SPDT, while the material spring back is enhanced as a proportional function of the ratio of hardness to the Young’s modulus. Considering the above two factors, the machinability of PMMA using SPDT is improved by cryogenic cooling and a better surface quality can be achieved. 

Cryogenic cooling has been widely attempted in machining materials including ceramics, polymers, metals, and alloys [29,30,31,32]. Nevertheless, no reports are available for the cryogenic cooling of PMMA during SPDT. In the previous reports, for example, the effect of cryogenic cooling on carbide–cobalt alloys mainly led to an increase in hardness compared to that at room temperature, while maintaining toughness, transverse rupture and impact strength, which can explain why the carbide tool materials are suitable for the cryogenic cooling method. Meanwhile, for some high-speed steels, cryogenic cooling does not affect hardness obviously, which means that cryogenic cooling will not affect their machining properties [32]. For PMMA, as tested in this work, cryogenic cooling is effective in increasing its hardness, even though its Tg is over 100 °C. The increase in hardness is effective in improving surface quality and workpiece precision during ultra-precision machining with optimized processing parameters. Accordingly, this work demonstrates that cryogenic cooling is effective for polymeric materials with a high Tg during ultra-precision machining, which opens a new avenue for manufacturing high-precision polymeric components. It contradicts the conventional wisdom that polymeric materials are soft and difficult to use as high-precision components compared to metal and alloys. The results in this work prove that it is feasible to fabricate high-precision polymeric components via cryogenic cooling.

## 4. Outlook

In this work, the effect of cryogenic cooling on the SPDT of PMMA is mainly studied from the view of material properties. During machining, the effects on tool wear, residual stress distribution, and chip formation can all contribute to the surface quality of the final workpiece. These effects will be studied in future work. Even though cryogenic cooling is helpful in assisting ultra-precision machining, some problems still need to be solved. One issue with cryogenic cooling is that the super-cold medium needs to be continuously supplied. The pipeline design and corresponding fixtures increase the cost of the final parts and consume time. In addition, the position of the output nozzle and volume flux have notable impacts on the machined parts. The above challenges took the authors plenty of time to address in this work. Not surprisingly, cryogenic cooling is generally used in research, but is not yet suitable for large-scale production. Further efforts need to be made before the cryogenic cooling process can be industrialized.

## 5. Conclusions

In this work, the machinability and surface properties of cryogenically cooled PMMA machined via SPDT were investigated. The cryogenic cooling was completed using liquid nitrogen. The optimal parameters for machining at room temperature and 0 °C were obtained using the Taguchi method. The form accuracy Pt and surface roughness Ra of the workpieces under optimal conditions were measured and compared. At room temperature, a Pt value of 291 nm and an Ra of 11 nm were obtained, while at 0 °C, a Pt value of 124 nm and an Ra of 6 nm were observed, displaying a significant improvement compared to the values at an ambient temperature. For the workpieces machined at room temperature, a spindle speed of 1500 rpm, a feed rate of 5 mm/min, and a cut depth of 4µm can be used to obtain lowest values of Pt and Ra. The optimal processing conditions for the SPDT of cryogenically cooled PMMA at 0 °C are a spindle speed of 2500 rpm, a feed rate of 10 mm/min and a cut depth of 2 µm. To reveal the mechanism of this process, the relationship between the temperature, material properties and machinability of PMMA was analyzed. The material property changes with temperature were studied via nanoindentation tests. As demonstrated by the experimental results, the hardness and Young’s modulus at 0 °C increased by 37% and 22% compared to those at room temperature, respectively. The change in mechanical properties is mainly attributed to the viscoelasticity’s variation with temperature. When temperature changed from 25 °C to 0 °C, the bulk material showed more elastic behavior with a decrease in tanδ, as verified by DMA tests. In addition, a model of the surface profile in SPDT was used to qualitatively explain the smoother surfaces at lower temperatures due to change in material properties. Decreasing the temperature can suppress sub-Tg relaxation, causing an enhancement of mechanical properties, which results in a better surface quality when using SPDT. It can be concluded that cryogenic cooling is effective in the ultra-precision machining of PMMA due to the material property changes caused by temperature. In future work, cryogenic machining can be attempted for materials like PMMA with a high Tg to achieve a high surface quality.

## Figures and Tables

**Table 1:** HV values of cryogenically cooled PMMA at different temperatures obtained using a micro hardness tester.

Temperature (°C)	Average HV (kgf/mm^2^)	Standard Deviation
0	30.2	0.09
5	26.4	0.12
10	22.2	0.10
15	21.4	0.11
20	19.8	0.03
25	20.2	0.07

**Table 2:** Factors and levels in the SPDT of PMMA.

Factors	Levels		
	Level 1	Level 2	Level 3
Spindle speed (rpm)	1500	2000	2500
Feed rate (mm/min)	5	8	10
Cut depth (µm)	2	4	8

**Table 3:** Comparison of three factors of the surface profile model at 25 °C and 0 °C.

Factors	0 °C	25 °C
*Rtew*(*x*)	=	
*w_r_*		>
*s_r_*	>	

## Data Availability

Data are contained within the article.
